# Supplementary material for: Perfect absorption in complex scattering systems with or without hidden symmetries
Source: Nat Commun. 2020 Nov 17;11:5826. doi: 10.1038/s41467-020-19645-5 (PMC7673030; doi:10.1038/s41467-020-19645-5)
Supplement: Supplementary file 1 — Supplementary Information [file 41467_2020_19645_MOESM1_ESM.pdf]

Supplementary Information for Perfect Absorption in Complex Scattering Systems with or without Hidden Symmetries

Lei Chen,[1,2] Tsampikos Kottos,[3] and Steven M. Anlage[1,2]

[1] *Quantum Materials Center, Department of Physics, University of Maryland, College Park, MD 20742, USA*
[2] *Department of Electrical and Computer Engineering, University of Maryland, College Park, MD 20742, USA*
[3] *Wave Transport in Complex Systems Lab, Department of Physics, Wesleyan University, Middletown, CT 06459, USA*

SUPPLEMENTARY NOTE 1: EXPERIMENTAL SETUP FOR S-MATRIX MEASUREMENT

The S-matrix measurement involves the VNA and the microwave graph (see Supplementary Figure 1). Calibration is done at the end of two test cables (see red lines in Supplementary Figure 1) where they are connected to the graph. The S-matrix of the experimental setup is measured under many settings of the variable attenuator.

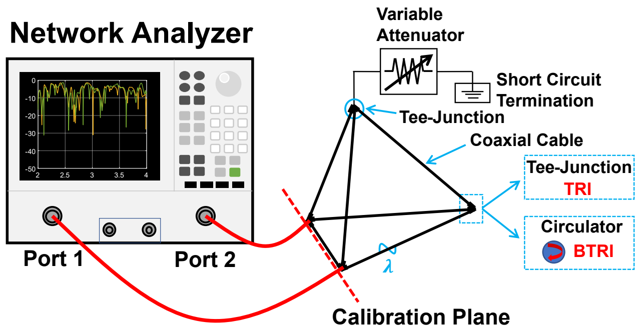

Supplementary Figure 1. **Schematic experimental setup of the S-matrix measurement.** The tetrahedral graph is formed by coaxial cables connected with Tee-junctions. One node of the graph is loaded with a voltage variable attenuator to provide parametric variation of the scattering system. One other node (blue dashed box) is made from either a Tee-junction (TRI) or a 3-port circulator (BTRI) to create a TRI system or a broken-TRI system, respectfully.

SUPPLEMENTARY NOTE 2: SIMULATION OF THE "ANTI-CPA" STATE

We introduce a new operator – the Absorption matrix $A \equiv \mathbb{1} - S^{\dagger}S$ to analyze the "Anti-CPA" state[1]. We point out that A is a Hermitian, positive semi-definite operator. The magnitude of its eigenvalues α span the interval [0,1] and the corresponding eigenvectors $|\alpha\rangle$ are orthogonal. It is easy to show that the eigenvector associated with the eigenvalue $\alpha_{\max} = 1$ is the CPA waveform that we have identified previously from the analysis of the zeroes of the S-matrix. It follows that the components of the eigenvector which is associated with the minimum eigenvalue $\alpha_{\min}$ provides the shape of the incident waveform which will lead to minimal absorption. We refer to such a scattering field as the "Anti-CPA" state. The extreme case of $\alpha = 0$ is associated with a scattering field that avoids completely the vertex where the attenuator is located. We verify this effect by observing the voltage profile and energy distribution in the system under "Anti-CPA" stimulus at the same frequency in the simulation (see Supplementary Figure 2a). In Supplementary Figure 2b, the voltage on each node is much smaller than the voltage at CPA state (compare with Fig. 4b of the main text), and the voltage on node 4 where the attenuator is attached is particularly small. Under this condition, the total power absorption ratio is only 0.13, and nearly no power is absorbed by the attenuator (Supplementary Figure 2c), which characterizes the "Anti-CPA" state. Nevertheless, there is a great deal of reactive power present in the system (compare with Fig. 4c of the main text).

SUPPLEMENTARY NOTE 3: QUALITY OF SIMULATION

We set up the simulation model by measuring the S-matrix of every individual component in the graph experiment, and import them as TOUCHSTONE file blocks into CST. The S-matrices are combined in the same topology as the graph of interest to create the simulation. An example comparison of the entire graph S-matrix (see Supplementary Figure 3) is made between the simulation result and the experimental measurement, when the applied voltage of the variable attenuator is 5.00 V. The determinant of the 2×2 S-matrix ($\det(S)$) is used for evaluating the quality of simulation for this TRI graph. Supplementary Figure 3 presents both the magnitude and phase of $\det(S)$ in a selected frequency range. From Supplementary Figure 3, the simulation results are in very good agreement with the experimental measurement, and can well characterize the CPA response.

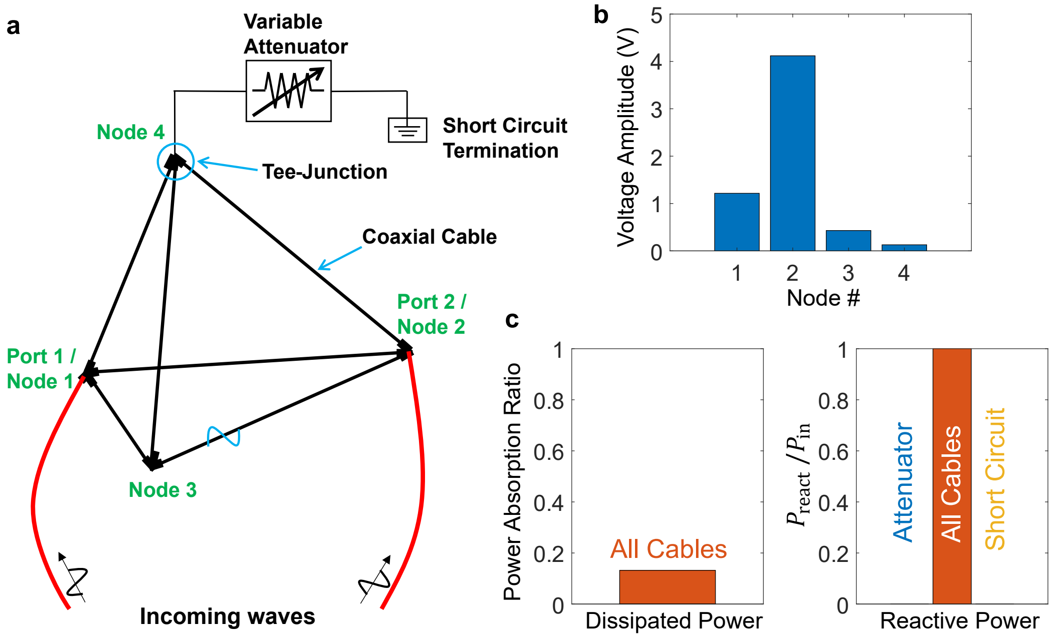

Supplementary Figure 2. **Voltage profile and power distribution of the "Anti-CPA" state in idealized simulation.** **a** | Schematic of the microwave graph with labeled ports under CPA condition at 2.2999 GHz in simulation. **b** | Voltage profiles of four nodes in the graph under the "Anti-CPA" condition. The voltage amplitude on Node 4, where the absorbing attenuator is attached, is much less than the voltage amplitude on other nodes. **c** | Power distribution among the graph components under the "Anti-CPA" condition. Left plot shows that very little power (less than 15%) are absorbed by the graph, and almost no power is dissipated by the attenuator. Right plot shows reactive power on the cables.

SUPPLEMENTARY NOTE 4: SWEPT MEASUREMENT OF THE CPA STATE IN THE QUARTER BOW-TIE BILLIARD

For the CPA state at $f = 14.2888$ GHz (see the blue line in Fig. 5 of the main text) in the quarter bow-tie billiard, we perform the other two independent parametric sweep measurements, following the same procedure as used in Figs. 3 and 6 of the main text. The amplitude sweep and phase sweep measurements shown in Supplementary Figure 4 demonstrate similar characteristic features, which demonstrate a CPA state in the two-dimensional quarter bow-tie billiard.

SUPPLEMENTARY REFERENCES

[1] H. Li, S. Suwunnarat, R. Fleischmann, H. Schanz, and T. Kottos, Random matrix theory approach to chaotic coherent perfect absorbers, Physical Review Letters **118**, 044101 (2017).

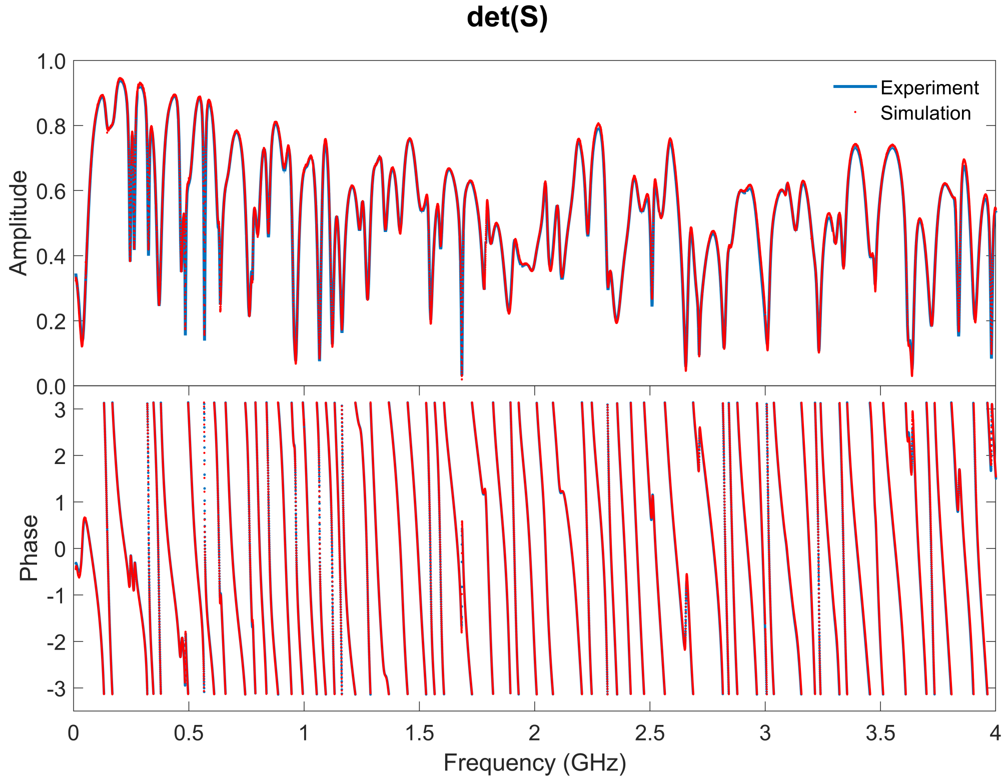

Supplementary Figure 3. **Comparison of S-matrix from simulation and experiment of the tetrahedral graph setup.** The amplitude and phase of $\det(S)$ are compared between the simulation and experiment of the TRI graph setup when the applied voltage of the variable attenuator is set to 5.00 V. The blue line represents the experimental measurement, and the red dots are the simulation data.

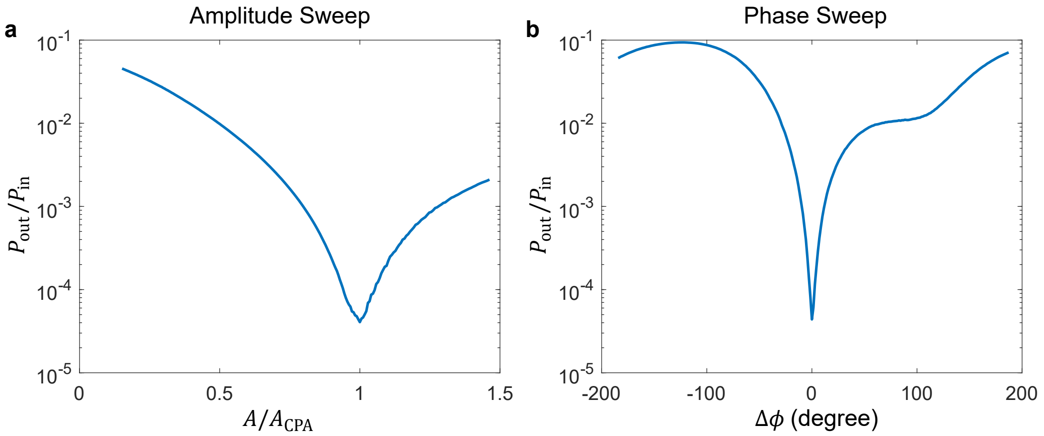

Supplementary Figure 4. **Evidence of CPA in the quarter bow-tie billiard under two independent parametric sweeps.** Measurements are performed for the CPA state at $f = 14.2888$ GHz in the quarter bow-tie billiard. Output to input power ratio obtained by changing the amplitude A at port 1 (**a**) and phase difference $\Delta\phi$ at port 2 (**b**) separately of the two excitation signals required for the CPA state. All experimental results are obtained by direct measurement of the input and output RF powers.
